# Psychosocial interventions and mental health in patients with cardiovascular diseases living in low and middle-income countries: A systematic review protocol

**DOI:** 10.1371/journal.pone.0271955

**Published:** 2022-07-28

**Authors:** Saima Hirani, Ambreen Gowani, Sehrish Sajjad, Megan Kennedy, Colleen M. Norris

**Affiliations:** 1 School of Nursing, Faculty of Applied Science, The University of British Columbia, Vancouver, Canada; 2 School of Nursing & Midwifery, Faculty of Health Sciences, The Aga Khan University, Karachi, Pakistan; 3 Health Sciences Library, University of Alberta, Edmonton, Canada; 4 Faculty of Nursing, Public Health, Faculty of Medicine and Dentistry, University of Alberta, Edmonton, Canada; Universiti Putra Malaysia, MALAYSIA

## Abstract

**Objective:**

The objective of this review is to evaluate the effectiveness of psychosocial interventions on mental health outcomes in adult patients with Cardiovascular Diseases (CVDs) living in low- and middle-income countries (LMICs).

**Introduction:**

Mental health issues are highly prevalent among patients with CVDs leading to poor disease prognosis, self-care/ management, and Quality of Life (QOL). In the context of LMICs, where the disease burden and treatment gap are high and resources are inadequate for accessing essential care, effective psychosocial interventions can make significant contributions for improving mental health and reducing mental health problems among patients who live with cardiovascular diseases.

**Inclusion criteria:**

This review will include studies published between 2010 and 2021 that evaluated the effect of psychosocial interventions on mental health outcomes (resilience, self-efficacy, QOL, depression and anxiety) on adult patients (aged ≥18 years) with any cardiovascular diseases using experimental and quasi experimental designs.

**Methods:**

The search will be conducted from the following databases: MEDLINE via OVID (1946—Present), EMBASE via OVID (1974 –Present), Cumulative Index for Nursing and Allied Health Literature (CINAHL) via EBSCOhost (1936—Present), PsycINFO via OVID (1806—Present), Scopus via Elsevier (1976—Present), and Cochrane Library via Wiley (1992—Present). Data will be critically appraised using standard tools and extracted by two reviewers and disagreement will be solved by the third reviewer. Meta-analysis will be performed, if possible, otherwise, data will be synthesized in narrative and tabular forms.

**Discussion:**

The findings of this review will provide a key insight into contextually relevant psychosocial interventions for promoting mental health of patients with CVDs living in LMICs. The review findings will be potentially useful for health care providers and researchers to implement such interventions not only for reducing the burden of mental health issues but also for improving the overall well-being among patients with chronic illnesses.

**Systematic review registration number:**

Prospero-CRD42020200773.

## Introduction

Cardiovascular diseases (CVDs) are the leading cause of mortality worldwide with the higher burden in the low- and middle-income countries (LMICs). The proportion of risk factors as well as the rate of premature mortalities due to CVDs is highest among the LMICs compared to some of the high-income populations of the world [1, 2] and is projected to rise at such an alarming rate that it can become catastrophic to the already stretched health care systems of LMICs. People in LMICs encounter CVDs at a much younger age compared to the rest of the world which compromises the most productive years of their lives as well as propel them to premature deaths [1]. CVDs intrinsically restrict patients’ physiological, psychological and social functioning [3]. The burden is further aggravated by the resource constrained health care systems in LMICs which are unable to meet the needs of the population due to lack of structured health financing and strong health care delivery models. This compels patients to bear their health care expenditure out of their pockets [4] and make them vulnerable to further stress. Consequently, patients residing in LMICs poorly adhere to self-care and timely health seeking behaviors. This contributes to high rates of preventable complications and mortalities [1].

In addition to premature mortalities, some socioeconomic determinants such as poverty, illiteracy, and lack of resources also contribute to increasing mental health issues among patients with cardiovascular disorders. Poor mental health has been known as an independent predictor of mortality and poor clinical prognosis among patients with CVDs [5–7]. Statistics suggest that more than 85% of the world population live in LMICs [8] and by 2030, depression and CVDs are going to be the third and fifth leading causes of disease burden in these countries respectively [9]. Depression and anxiety are found to have strong associations with mortality among patients with Heart Failure (HF) and Coronary Artery Disease (CAD) [3, 5]. The risk is higher among patients who remain clinically undiagnosed with mental health disorders but silently continue to suffer from its devastating effects such as; inability to adopt lifestyle modifications, poor adherence to medication regimen, and poor judgment to call for help [10]. On the other hand, patients with optimum mental health have shown better progress with CVDs by improving their lifestyle and self-management behaviors across the spectrum of CVDs [10]. Management of CVDs is a shared decision making between patients and health care providers demanding a commitment to a healthy lifestyle, which is only possible if a person is in a positive outlook and has capacity to take care of him/herself. It is, therefore, imperative to invest in the mental health promotion of patients to enhance their overall well-being so they can lead better lives [11].

Various concepts contribute to the understanding of mental health, however, resilience, self-efficacy and quality of life (QOL) are considered some of the key indicators of positive mental health [12] and have been associated with positive health outcomes in patients with CVDs [13, 14]. The current understanding of resilience explains it as a capacity and process that allows individuals to deal with stressors and sustain their functionality [15]. Self-efficacy has been described as an ability or belief of individuals to execute behavior that are required to produce effective outcomes [16]. QOL is another multifaceted element of mental health that refers to individual’s perceived satisfaction with their position in life in relation to their goals and expectations [17]. Research shows a significant positive association among these variables and a negative correlation with depression among patients with cardiac diseases [18, 19].

Psychosocial interventions are generally defined as non-pharmacological measures targeted on psychological and social domains to improve mental health including recovery, social skills, daily functioning, quality of life and community’s attitudes towards individuals with mental health conditions [20]. Evidence based practice guidelines shared by Nursing Intervention Classification and the National Institute of Health and Care Excellence also include psychosocial interventions that have demonstrated positive outcomes in patients with CVDs and reduced their depression [21, 22].

Several systematic reviews on interventions including but not limited to cognitive behavioral therapy, individual and group counseling, mindfulness, social support and educational interventions have shown positive effects such as reduced anxiety and depression, improved quality of life, increased social support, and enhanced self-esteem and resilience among patients with CVDs [23–26]. A number of reviews also concluded to include psychosocial interventions in cardiac rehabilitation programs given their positive effects on emotional and psychological well-being [27, 28]. However, most studies have predominantly focused on improving depressive symptoms and contained low quality evidence. None of these have focused on promoting resilience, self-efficacy, and QOL in the context of LMICs.

Evidence suggest several studies that implemented contextually relevant psychosocial interventions in a variety of locations including hospitals, rehabilitation centers and community settings and have improved mental health outcomes among patients with CVDs [29–31]. For instance, some studies trained community health workers instead of health care professionals to deliver interventions, which is a very unique and adaptable approach to other low-resource settings of developing countries [32, 33]. However, to date, there is no systematic review available which summarizes and evaluates the effectiveness of psychosocial interventions on mental health outcomes of patients with CVDs in LMICs.

### Aim of the review

The aim of this proposed review is to evaluate the effectiveness of psychosocial interventions (compared to other psychosocial interventions or no interventions) on mental health outcomes in patients with cardiovascular diseases living in low- and middle-income countries. The findings of the review will be useful for practitioners, researchers, and policymakers to implement evidence informed care for promoting mental health of patients with CVDs living in LMICs. The findings may also have potential to advocate for introducing a holistic and integrated model of care for patients living with chronic illnesses in resource constrained settings. The proposed review will aim to answer the following question:

What is the effectiveness of psychosocial interventions on mental health outcomes in patients with cardiovascular diseases living in low-and middle-income countries?

## Methods

The proposed systematic review will be guided by the Preferred Reporting Items for Systematic Reviews and Meta-Analyses Protocols (PRISMA-P) checklist [34] (S1 Checklist). The review is registered in PROSPERO (reg number: CRD42020200773).

### Participants

The proposed review will include studies which were conducted on adult participants aged ≥18 years, residing in LMICs, suffering from any form of CVDs. The spectrum of these diseases encompasses: Rhythm disorders (tachycardia, bradycardia, atrial fibrillation, atrial flutter, patients with pacemakers or intra cardiac defibrillators), CAD (all types of angina, myocardial infarction, ischemic heart disease, acute coronary syndrome, patients with angioplasty or Coronary Artery Bypass Graft (CABG), HF (systolic or diastolic heart failure with preserved or reduced function), structural heart disease (congenital heart defects or valvular disease), cardiomyopathies (ischemic or non-ischemic). We will also include studies that are conducted on patients with hypertension (systolic or diastolic), given its strong association with CVDs. Participants may or may not be suffering from the mental illness at the baseline. We will also include studies with co-existence of CVDs with other diagnosis, provided that separate analysis on patients with CVDs is reported. However, we will exclude studies which are exclusively conducted on patients who are at risk of heart diseases. For instance, patients with metabolic syndrome characterized by dyslipidemia, obesity, diabetes mellitus, unhealthy lifestyle (sedentary life style, smoking, unhealthy eating habits, alcohol consumption), or renal problems.

### Intervention(s)

This review will consider studies that evaluate psychosocial interventions i.e. all non-pharmacological interventions focusing on psychological and social domains to promote positive mental health i.e. improving resilience, self-efficacy, and quality of life; and reducing depression and anxiety among patients with cardiovascular diseases in LMICs. Included interventions may differ in nature, content, duration, format (e.g. individual and group), delivery mode (e.g. face to face, e-learning, and blended), strategy (delivered by health care professionals and trained non-health care trainers) and setting (hospitals/clinics, rehabilitation, and community).

### Comparator(s)

We will include all studies that have compared psychosocial interventions with comparative control groups, multiple intervention arms (allowing comparison of different types of psychosocial interventions) or no control group. However, the eligibility of interventions to be included in the review will only be considered if the effect of psychosocial interventions is measured independently from co-interventions. We will exclude those interventions which only evaluated physiological outcomes related to cardiovascular diseases and treatments (e.g. exercise, diet, smoking, medicines etc.).

### Outcomes

The review will consider studies which include the following mental health outcomes: resilience, self-efficacy, and quality of life (QOL will include general QOL as well as heath related QOL). The pre-specified secondary outcomes for this review are; depression and anxiety. The duration of the study, points of measurement (before/after, only after intervention and follow-up measures), and instruments used to measure the same outcome might vary. Outcomes can be measured using standardized or self-developed instruments.

### Types of studies

We will consider both randomized controlled trials (RCTs) and quasi-experimental studies for the review. Studies published in English language will be included in the review. Studies published between 2010 and 2021 will be selected to capture recent and relevant evidence of the effectiveness of the psychosocial interventions on mental health of patients with CVDs in LMICs.

### Search strategy

This systematic review will be conducted and reported in adherence with the Preferred Reporting Items for Systematic Reviews and Meta-Analyses (PRISMA) statement and checklist [34]. A search strategy will be developed by an experienced health sciences librarian (MK), who is familiar with systematic review methodology and systematic, advanced searching of health sciences databases. The research team will work with MK to identify relevant vocabulary for the following concepts: 1) Psychosocial interventions, including psychotherapy and other psychotherapeutic interventions such as counseling, cognitive behavior therapy, psychoeducation, social support and mindfulness practices; 2) Cardiovascular diseases. The search will be limited geographically to low-and-middle income countries designated by World Bank data [8] (current as of August 2020). A combination of controlled vocabulary (wherever available) and natural language keywords will be used to construct the search strategies in each database. A preliminary search was conducted in Medline in order to determine feasibility of this review (S1 File).

### Information sources

The search will be conducted from the following information sources:

- Medline via OVID (1946—Present)
- EMBASE via OVID (1974—Present)
- Cumulative Index for Nursing and Allied Health Literature (CINAHL) via EBSCOhost (1936—Present)
- PsycINFO via OVID (1806—Present)
- Scopus via Elsevier (1976—Present)
- Cochrane Library via Wiley (1992—Present)

### Study selection

We will collate and upload all identified citations into Covidence. Duplicates will be removed. Two reviewers will independently screen study titles and abstracts and select studies based on inclusion criteria. The citations will be marked as potentially suitable for inclusion, not suitable, or uncertain. Reasons for studies not meeting the inclusion criteria will be recorded. Full text of potentially suitable or uncertain articles will be retrieved. Discrepancies between the reviewers at each stage of the study selection will be arbitrated by a third reviewer. The results obtained from the search will be reported and presented in the Preferred Reporting Items for Systematic Reviews and Meta-analyses (PRISMA) flow diagram [34].

### Assessment of methodological quality

Selected studies will be critically assessed by two independent reviewers for the methodological quality prior to inclusion in the review using the standardized critical appraisal instruments from the JBI for experimental and quasi-experimental studies [35]. RCTs will be assessed for methods of randomization, treatment allocation, blinding, risk of bias, homogeneity at baseline, intent to treat analysis, use of valid measurement tools, and appropriate statistical analysis. For quasi-experimental studies, the assessment will be made for independent and dependent variables, intervention allocation/other exposure, control group or no control group, multiple point of measurements (baseline, post intervention, follow-up measurement), reliable outcome measurements and appropriate statistical analysis. We will contact study authors to request any missing or additional information for clarification. We will resolve any disagreements that arise between the reviewers through discussion and arbitration by a third reviewer. The findings obtained from the critical appraisal will be presented in tabular and narrative forms.

All studies, regardless of the results of their methodological quality will undergo data extraction and synthesis (if possible). The findings of the critical appraisal would also serve as evidence and will be explicitly presented in the review.

### Data extraction

Two reviewers will independently extract data using Joanna Briggs Institute data extraction tool [36]. The extracted data will include information about study design, participants’ demographic and clinical profile, location and setting of the study, study methods, interventions (what, who and how), details of control group, and outcomes of significance to the review objective. Both reviewers will verify the extracted data together from the original published papers. We will resolve any inconsistencies via discussion and arbitration by a third reviewer. We will contact corresponding authors of the studies to request additional or missing data, if required.

### Data synthesis/analysis

We will pool studies with statistical meta-analysis, wherever possible. Statistical analysis will be carried out by using RevMan 5.1. We will report effect sizes as odds ratios for dichotomous data and standardized mean differences or mean differences for continuous data and their 95% confidence intervals. For studies that only report dichotomous outcomes, we will estimate the effect by converting the standard error of the log odds ratio to the standard error of a SMD using RevMan. We will assess clinical heterogeneity by taking into consideration clinical characteristics of study participants; whereas, using the chi-squared test statistics we will determine statistical heterogeneity within studies. We will explain reasons for heterogeneity and perform sensitivity analysis wherever the data permits. Given the differences in the outlook of mental health among men and women in LMICs, if possible, we aim to study the effect of psycho-social interventions on cardiac patients’ mental well-being across sex and gender. If possible, we also intend to examine the differences in the effect on interventions delivered by health care professionals versus community workers. We will use multi-variable regression for the individual level data to perform above mentioned subgroup analyses.

We will perform sensitivity analysis to assess the effect of removing any study with high risk of bias from the analysis and also to determine the effect of removing quasi experimental studies from the analysis. If the included studies are at least ten for meta-analysis, we will assess publication bias by developing funnel plots of effect sizes as a visual presentation. We will also perform statistical tests for funnel plot asymmetry (Egger test, Begg test, Harbord test) where appropriate. Where statistical pooling is not possible, the findings will be presented in a narrative form including tables and figures to aid in data presentation, where appropriate.

The overall quality of the evidence for each outcome will be assessed using the Grading of Recommendations, Assessment, Development, and Evaluation (GRADE) guidelines [37].

## Discussion

Promoting mental health of patients with cardiovascular diseases is critical for their overall well-being. Addressing mental health needs of these patients is challenging in the context of resource constrained health care settings like LMICs. In this protocol, we aim to identify effectiveness of psychosocial interventions on mental health outcomes of patients with CVDs.

The findings of this review will provide a key insight into what interventions are effective in the context of LMICs, where health care resources are scarce. The findings will also inform researchers about key research gaps and future research directions.

We anticipate some limitations that we would like to acknowledge. First, for this review we are primarily interested in positive outcomes related to mental health. Since mental health is a multi-layered construct, there could be several indicators or variables that represent this phenomenon. For this review, our primary outcomes are resilience, self-efficacy, QOL and our secondary outcomes include improvement in depression and anxiety which are the most common mental health concerns among patients with CVDs. In order to have a focused scope of this review, we will not include other mental health variables. Second, this review will only include studies published in English language, this may not allow us to include knowledge published in other languages. However, we think a significant number of studies are available in English.

We will disseminate our findings through a peer reviewed publication and also through presentations in conferences and academic and stakeholders’ meetings.

## Supporting information

S1 Checklist PRISMA-P (Preferred Reporting Items for Systematic review and Meta-Analysis Protocols) checklist: Recommended. (DOC)

S1 File Search strategy. (DOCX)
