# Supplementary material for: Psychosocial interventions and mental health in patients with cardiovascular diseases living in low and middle-income countries: A systematic review protocol
Source: PLoS One. 2022 Jul 28;17(7):e0271955. doi: 10.1371/journal.pone.0271955 (PMC9333280; doi:10.1371/journal.pone.0271955)
Supplement: S1 File — (DOCX) [file pone.0271955.s002.docx]

**S2 Appendix: Preliminary Search strategy**

Database: OVID Medline(R)

| **Line number** | **Search terms** | **Number of results** |
| --- | --- | --- |
| 1 | Psychoso*.mp. | 114996 |
| 2 | ((social or peer or family or parent* or spous* or community) adj3 support*).mp. | 119718 |
| 3 | community networks/ or social support/ or psychosocial support systems/ | 77485 |
| 4 | Self-Help Groups/ | 9103 |
| 5 | exp psychotherapy/ or exp animal assisted therapy/ or exp behavior therapy/ or exp cognitive behavioral therapy/ or exp relaxation therapy/ or exp feedback, psychological/ or exp hypnosis/ or exp suggestion/ or exp psychoanalytic therapy/ or exp psychotherapeutic processes/ or exp abreaction/ or exp transference, psychology/ or socioenvironmental therapy/ or exp milieu therapy/ or exp psychotherapy, group/ | 195698 |
| 6 | exp counseling/ or exp directive counseling/ | 44003 |
| 7 | exp mind-body therapies/ or exp breathing exercises/ or spirituality/ or exp Spiritual Therapies/ or exp religion/ | 111791 |
| 8 | (psychotherap* or "psycho-therap*" or psycholog* or "motivational interview*" or "motivational enhancement therap*" or (family adj2 therap*) or (behavio?r* adj2 therap*) or (cognitive adj2 therap*) or (psychodynamic adj2 therap*) or (relaxation adj2 therap*) or "guided imagery" or psychiatr*).mp. | 1744553 |
| 9 | ((group or individual) adj2 (therapy or counsel?ing)).mp. | 23550 |
| 10 | (psychoeducat* or "psycho-educat*").mp. | 6559 |
| 11 | (mindful* or meditat* or prayer* or praying or spiritual* or religion* our religious or faith or faiths or theolog* or "belief system*").mp. | 46870 |
| 12 | Mental Health/ or ("mental* health*" or "well-being" or "wellbeing").mp. | 273183 |
| 13 | or/1-12 | 2047473 |
| 14 | exp cardiovascular diseases/ or exp cardiovascular abnormalities/ or exp heart defects, congenital/ or exp coronary vessel anomalies/ or exp dextrocardia/ or exp heart septal defects/ or exp "transposition of great vessels"/ or exp vascular malformations/ or exp arteriovenous malformations/ or exp vascular fistula/ or exp cardiovascular infections/ or exp endocarditis, bacterial/ or exp tuberculosis, cardiovascular/ or exp heart diseases/ or exp arrhythmias, cardiac/ or exp cardiac conduction system disease/ or exp cardiomegaly/ or exp cardiomyopathies/ or exp endocarditis/ or exp heart arrest/ or exp heart failure/ or exp heart neoplasms/ or exp heart rupture/ or exp heart valve diseases/ or exp myocardial ischemia/ or exp pericarditis/ or exp ventricular dysfunction/ or exp ventricular outflow obstruction/ or vascular diseases/ or exp aneurysm/ or exp angiodysplasia/ or exp angiomatosis/ or exp angioedema/ or exp aortic diseases/ or exp arterial occlusive diseases/ or exp cerebrovascular disorders/ or exp compartment syndromes/ or exp diabetic angiopathies/ or exp "embolism and thrombosis"/ or exp hemostatic disorders/ or exp hypertension/ or exp hypotension/ or exp peripheral vascular diseases/ or exp reperfusion injury/ or exp telangiectasis/ or exp thoracic outlet syndrome/ or exp varicose veins/ or exp vasculitis/ or exp venous insufficiency/ | 2385067 |
| 15 | (((cardiovascular or "cardio-vascular" or "cardio vascular" or cardiac or heart* or circulat* or coronary or myocardial) adj3 (disease* or infection* or illness* or disorder* or condition* or problem* or failure* or arrest or attack* or infarction* or thrombosis)) or angina or tachycardia).mp. | 1177857 |
| 16 | (hypertension or "hyper-tension" or "blood pressure").mp. | 780793 |
| 17 | (hypotension or "hypo-tension").mp. | 67702 |
| 18 | 14 or 15 or 16 or 17 | 2981899 |
| 19 | Developing Countries/ | 74851 |
| 20 | ("low-and-middle-income countr*" or LMIC or LMICs or "developing countr*" or "developing nation*" or Afghanistan or Albania or Algeria or "American Samoa" or Angola or Argentina or Armenia or Azerbaijan or Bangladesh or Belarus or Belize or Benin or Bhutan or Bolivia or "Bosnia and Herzegovina" or Botswana or Brazil or Bulgaria or "Burkina Faso" or Burundi or "cabo verde" or Cambodia or Cameroon or "Cape Verde" or "Central African Republic" or Chad or China or Colombia or Comoros or Congo or "Costa Rica" or "Cote d'Ivoireor Ivory Coast" or Cuba or Djibouti or Dominica or "Dominican Republic" or Ecuador or Egypt or "El Salvador" or Eritrea or Eswatini or Ethiopia or Fiji or Gabon or Gambia or Georgia or Ghana or Grenada or Guatemala or Guinea or "Guinea-Bissau" or Guyana or Haiti or Honduras or India or Indonesia or Iran or Iraq or Jamaica or Jordan or Kazakhstan or Kenya or Kiribati or Korea or Kosovo or Kyrgyz or Lao or Lebanon or Lesotho or Liberia or Libya or Madagascar or Malawi or Malaysia or Maldives or Mali or "Marshall Islands" or Mauritania or Mauritius or Mexico or "Federated States of Micronesia" or Moldova or Mongolia or Montenegro or Morocco or Mozambique or Myanmar or Namibia or Nepal or Nicaragua or Niger or Nigeria or "north macedonia" or Pakistan or "Papua New Guinea" or Paraguay or Peru or Philippines or Romania or Rwanda or Samoa or "Sao Tome and Principe" or Senegal or Serbia or Seychelles or "Sierra Leone" or "Solomon Islands" or Somalia or "South Africa" or "Sri Lanka" or "Saint Lucia" or "Saint Vincent and the Grenadines" or Sudan or Suriname or Swaziland or Syria* or Tajikistan or Tanzania or Thailand or "Timor-Leste" or Togo or Tonga or Tunisia or Turkey or Turkmenistan or Tuvalu or Uganda or Ukraine or Uzbekistan or Vanuatu or Venezuela or Vietnam or "West Bank and Gaza" or Yemen or Zambia or Zimbabwe).ti,ab,kw,kf. | 1146047 |
| 21 | 19 or 20 | 1170257 |
| 22 | (((intervention* or experimental or "cross-over" or crossover or placebo) adj2 (study or studies or trial* or group*)) or "randomi?ed control* trial*" or RCT or RCTs or "clinical trial*").ti,ab,pt. | 1465330 |
| 23 | 13 and 18 and 21 and 22 | 608 |
